# Bio-Based Fertilizers from Waste: Nutrient Recovery, Soil Health, and Circular Economy Impacts

**DOI:** 10.3390/toxics14010090

**Published:** 2026-01-19

**Authors:** Moses Akintayo Aborisade, Huazhan Long, Hongwei Rong, Akash Kumar, Baihui Cui, Olaide Ayodele Oladeji, Oluwaseun Princess Okimiji, Belay Tafa Oba, Dabin Guo

**Affiliations:** 1 School of Civil Engineering, Guangzhou University, Guangzhou 510006, China; 2 School of Architectural Engineering, Guangzhou Institute of Science and Technology, Guangzhou 510540, China; 3 College of Plant Protection, Northwest A&F University, Xianyang 712100, China; 4 Department of Environmental Management, Faculty of Environmental Sciences, Lagos State University, PMB, Lagos 102101, Lagos State, Nigeria; oluwaseun.okimiji@lasu.edu.ng; 5 College of Natural Science, Arba Minch University, Arba Minch P.O. Box 21, Ethiopia

**Keywords:** bio-based fertilisers, waste valorisation, nutrient recovery, soil health, circular economy, sustainability assessment

## Abstract

Bio-based fertilisers (BBFs) derived from waste streams represent a transformative approach to sustainable agriculture, addressing the dual challenges of waste management and food security. This comprehensive review examines recent advances in BBF production technologies, nutrient recovery mechanisms, soil health impacts, and the benefits of a circular economy. This review, based on an analysis of peer-reviewed studies, demonstrates that BBFs consistently improve the physical, chemical, and biological properties of soil while reducing environmental impacts by 15–45% compared to synthetic alternatives. Advanced biological treatment technologies, including anaerobic digestion, vermicomposting, and biochar production, achieve nutrient recovery efficiencies of 60–95% in diverse waste streams. Market analysis reveals a rapidly expanding sector projected to grow from $2.53 billion (2024) to $6.3 billion by 2032, driven by regulatory support and circular economy policies. Critical research gaps remain in standardisation, long-term performance evaluation, and integration with precision agriculture systems. Future developments should focus on AI-driven optimisation, climate-adaptive formulations, and nanobioconjugate technologies.

## 1. Introduction

Global agricultural systems face unprecedented challenges in meeting food security demands while minimising environmental impacts, resulting in a critical need for sustainable nutrient management solutions [1]. Global municipal solid waste generation reaches approximately 2.01 billion tonnes annually, while agricultural residue production exceeds 5 billion tonnes of crop residues plus 9.26 billion tonnes of livestock manure, and synthetic fertiliser production consumes substantial fossil fuel resources and contributes significantly to greenhouse gas emissions [2,3]. This convergence of waste generation and nutrient demand present transformative opportunities for circular economy approaches that convert organic waste streams into valuable bio-based fertilisers [4].

Bio-based fertilisers represent a fundamental paradigm shift from linear “take-make-dispose” models to circular systems that recover nutrients, enhance soil health, and reduce environmental burdens [5]. Unlike synthetic fertilisers, which provide readily available nutrients through energy-intensive industrial processes, bio-based alternatives leverage biological transformation processes to convert organic waste into slow-release nutrient sources, while simultaneously improving soil structure, microbial diversity, and carbon sequestration potential [6]. With vermicomposting achieving 1.5× nitrogen recovery improvements compared to traditional composting methods [7].

The global bio-based fertiliser market demonstrates a remarkable growth trajectory, expanding from $1.38 billion in 2024 to a projected $2.83 billion by 2030, at a compound annual growth rate of 12.8% [8]. This expansion is driven by increasing regulatory restrictions on synthetic fertilisers, rising consumer demand for organic products, and the growing recognition of the critical role of soil health in agricultural sustainability [9,10]. However, successful implementation requires addressing technical challenges, including quality standardisation, shelf-life limitations, and variable field performance under diverse environmental conditions [11].

The environmental benefits of bio-based fertilisers extend beyond nutrient provisioning to include soil ecosystem restoration, carbon sequestration and reduced pollution potential [12]. Meta-analyses indicate that organic fertilisation systems increase soil organic carbon by 19% while enhancing microbial diversity indices compared to synthetic fertiliser regimes [1]. These improvements translate into enhanced ecosystem services, including water retention, erosion control, and climate resilience, providing long-term agricultural and environmental benefits [5].

This comprehensive review synthesises current knowledge on bio-based fertiliser production from waste streams and examines conversion technologies, characterisation parameters, soil health impacts, economic viability, and regulatory frameworks. We analysed peer-reviewed literature from 2015 to 2025 to provide current perspectives on technological advances, market trends, and implementation challenges. This review aims to inform researchers, policymakers, and industry stakeholders about the opportunities and barriers to scaling bio-based fertiliser systems within circular economic frameworks.

## 2. Types of Waste Materials for Bio-Based Fertiliser Production

### 2.1. Agricultural Residues

Agricultural residues constitute heterogeneous biomass categories with distinct compositional profiles that determine bio-based fertiliser suitability. Global crop residue generation exceeds 5.0 billion tonnes annually (2024), with cereal straws contributing 1.3–1.5 billion tonnes, representing continued growth of 1.8% CAGR since 2001, representing 70% of total agricultural residue availability [13,14,15]. Table 1 presents a comprehensive quantitative assessment of global agricultural waste generation across 17 waste categories, delineating annual production volumes, principal producing nations, and current utilisation rates derived from FAO Statistical Yearbook 2024 and USDA Foreign Agricultural Service data.

The spatial distribution of agricultural waste generation exhibits pronounced heterogeneity reflecting divergent cropping systems, climatic conditions, and agricultural intensification patterns. Asia dominates global crop residue production, accounting for 47% of total generation, primarily attributable to intensive rice-wheat systems in China (650–700 Mt annually) and India (686 Mt from top 10 crops alone) [19,20]. The United States contributes 400–450 Mt annually, with corn stover representing the predominant stream (196–250 Mt) [21]. Brazil’s agricultural waste profile is uniquely characterised by sugarcane bagasse (95–105 Mt) and soybean residues (85–95 Mt), with the sugarcane-ethanol industry generating 360 billion litres of vinasse annually as a potassium-rich liquid waste stream [22,23].

Within the European Union, agricultural waste generation totals approximately 700 Mt annually, with significant inter-member state variation [24]. France leads in cereal straw production (35–40 Mt), while Spain and Italy dominate olive and citrus residue generation. The Nordic and Baltic regions (addressed in Section 2.6) present unique challenges due to climatic constraints but also opportunities for integrated forest-agricultural residue systems. Sub-Saharan Africa generates an estimated 280–320 Mt of crop residues annually, with maize, cassava, and sorghum as primary contributors, though systematic quantification remains limited [25].

Compositional analysis reveals substantial variation across crop species: [26,27,28]. Nitrogen content variations critically affect the choice of processing pathways, with cereal straws containing 0.3–0.8% nitrogen (C:N ratios 60–120:1), leguminous residues exhibiting 0.8–1.8% nitrogen (C:N ratios 25–45:1), and pruning wastes demonstrating intermediate values of 0.5–1.2% nitrogen (C:N ratios 40–80:1) [29,30,31]. These compositional parameters directly determine optimal processing technologies, with high C:N ratio materials requiring nitrogen supplementation for biological processing while low C:N ratio feedstocks enable direct composting applications [32,33]

Beyond conventional cereal residues, emerging and underutilised agricultural feedstocks demonstrate substantial potential for bio-based fertiliser production. Insect frass, derived from black soldier fly (*Hermetia illucens*) and mealworm (*Tenebrio molitor*) farming operations, exhibits exceptional nutrient profiles with 2.5–4.8% nitrogen, 1.2–2.1% phosphorus, and 1.8–3.2% potassium, coupled with beneficial chitin content (5–8%) that enhances plant disease resistance [34]. Global insect farming generates approximately 250,000–350,000 tonnes of frass annually (2024), reflecting 15–28% CAGR since 2015 and projected to reach 1.2 million tonnes by 2030 under current expansion trajectories [18].

Oilseed crop residues constitute another substantial feedstock category. Sunflower husks, representing 20–25% of seed weight with global generation > 8 million tonnes annually, contain 1.0–1.5% nitrogen and demonstrate excellent bulking properties for composting applications [35]. Rapeseed residues, including stems and pods, generate 3–4 tonnes ha^−1^ with C:N ratios of 35–45:1, requiring nitrogen supplementation for optimal biological processing [36].

Leguminous root biomass, incorporating nitrogen-fixing nodules, provides unique advantages through elevated nitrogen content (2.5–3.8%) derived from biological nitrogen fixation, contributing 50–300 kg N ha^−1^ to subsequent crops [37]. Camellia oleifera seeds residue, demonstrate particular efficacy in biochar production, yielding products with big surface areas [38].

#### Quantitative Assessment of Priority Feedstocks for Bio-Based Fertiliser Production

The specific feedstocks highlighted for bio-based fertiliser development demonstrate substantial but regionally concentrated generation patterns requiring targeted valorisation strategies.

Rice husks and straw collectively represent one of the largest underutilised agricultural waste streams globally. Annual rice husk production reaches 120–160 Mt (constituting 20–25% of paddy weight), while rice straw exceeds 731 Mt globally [39]. However, utilisation rates remain critically low—over 100 Mt of rice straw undergoes open-field burning annually, particularly in South and Southeast Asia, contributing to severe air quality degradation and nutrient loss. China’s progressive straw burning bans, implemented since the 1990s, have reduced burning from 27% to 2–5% of generated straw, with field return increasing from 46% (2009) to 52% (2019) [40].

Sunflower residues (husks, stalks, and heads) total 60–70 Mt annually, with Russia (17–20 Mt) and Ukraine (14–18 Mt) accounting for over 50% of global production [41]. The 2022 disruption to Ukrainian agricultural exports highlighted the strategic importance of these residue streams for European bio-based fertiliser supply chains.

Buckwheat production residues represent a smaller but nutritionally distinctive waste stream, with global generation of 2–3 Mt annually. Russia dominates production (52%, 1.15–1.22 Mt), followed by China (23%) and minor contributions from Japan, Poland, and the United States [42]. Buckwheat husks constitute 18–26% of grain weight and are characterised by exceptionally high potassium (4.56–38.63% K_2_O in ash) and silica content (18–22%), rendering them particularly suitable for biochar and ash-based fertiliser production [43].

Lupine residues (*Lupinus* spp.) generate 3–5 Mt annually, with Australia contributing 58.5% of global production (predominantly narrow-leafed lupine, *L. angustifolius*) and Europe accounting for 32.4%, led by Poland (~500,000 tonnes) and Germany (150,000–200,000 tonnes) [41,44]. The nitrogen-fixing capacity of lupine (100–300 kg N ha^−1^ year^−1^) positions lupine residues as high-value green manure feedstocks requiring minimal processing for agricultural application [45].

Insect frass, particularly from black soldier fly (*Hermetia illucens*) and mealworm (*Tenebrio molitor*) production facilities, represents an emerging waste stream with exponential growth trajectory. Current global production reaches 250,000–350,000 tonnes annually, with frass output approximately 40 times larger than harvested insect biomass [18,34,46]. The European insect farming sector, supported by EU Novel Food Regulation approvals, projects 28% annual growth through 2030, with frass production anticipated to reach 1.2 million tonnes annually [46].

### 2.2. Food Waste Characterisation and Processing

Food waste is a high-value feedstock with a moisture content of 50–85% and rich nutritional profiles, including proteins, carbohydrates, and biodegradable compounds [47]. Processing challenges include variable composition, potential acidification during anaerobic digestion, and contamination risks, which require careful quality control [48].

Fruit and vegetable waste contains 30–50% edible parts, with peels rich in polysaccharides, proteins, fibres, and pectin that are suitable for composting and fermentation [49]. Kitchen waste processing through multiple routes, including dried pellets with anaerobically effective microorganisms, demonstrates 20–40% higher plant yields at dosages > 120 kg N ha^−1^ compared to conventional fertilisers [50].

Advanced valorisation technologies show remarkable potential, with food waste-derived BBFs achieving superior performance over mineral fertilisers in cool seasons [51]. These systems are particularly effective in urban agriculture applications, where local waste streams can be processed into high-quality fertiliser products [52].

### 2.3. Animal Manure Properties and Optimisation

Animal manure provides established feedstocks with well-defined nutrient profiles, although it requires optimisation for modern agricultural applications. Cattle manure typically exhibits imbalanced C/N ratios for optimal anaerobic digestion, necessitating co-digestion with high-carbon substrates to enhance its biogas potential [53]. Processing parameters favour thermophilic conditions (55–60 °C) for pathogen elimination and nutrient conservation [54].

Poultry manure has superior characteristics, with a higher pH and extractable phosphorus content compared to other manures. Poultry litter-derived biochar exhibits exceptional liming potential, with application rates of 5–15 t ha^−1^, depending on soil conditions [55]. Recent research has demonstrated conversion factors for predicting heavy metal concentrations in Belgian manure-derived digestates based on dry matter content, addressing quality control concerns [56].

Process optimisation includes the plasma treatment of digestates that capture reactive nitrogen from the atmosphere, creating nitrite and nitrate forms while reducing methane and ammonia emissions during storage [57]. These advanced treatments address the challenges of traditional manure management while enhancing its fertiliser value.

### 2.4. Industrial Organic Waste Streams

Industrial organic waste presents diverse opportunities with varying processing requirements and quality characteristics. Brewery waste exhibits high nitrogen (420.25 kg ha^−1^) and potassium (840 kg ha^−1^) content, with 60% total organic matter content in digestate [58]. Processing achieves 60% organic matter conversion to biogas within 15 days; however, heavy metal monitoring remains essential [59].

Brewery waste generation demonstrates significant regional variation [60,61]. The protein content variation (18–26% dry basis) directly influences downstream processing selection, with higher protein fractions favouring anaerobic digestion while lower concentrations optimise composting efficiency [60].

Paper mill sludge contains cellulosic organic matter suitable for composting with enhanced nutrient profiles, including nitrogen, phosphorus, and potassium [62]. Tank fermentation with straw, corncob, and mushroom residue demonstrates high-quality resource potential for organic fertiliser development [63].

Sugarcane vinasse is an innovative feedstock for mixed microbial inoculant production, generating three-fold higher fungal biomass than standard media, while reducing toxicity and improving soil characteristics [22]. This approach demonstrates the transformation of industrial waste into specialised biofertiliser products with enhanced microbial functionality.

### 2.5. Municipal Solid Waste Organic Fractions

Municipal solid waste organic fractions demonstrate compositional complexity. Recent meta-analyses encompassing 47 global cities reveal moisture content ranges of 45–85%, with developed nations trending toward lower values (45–65%) due to enhanced source separation, while developing regions exhibit higher moisture (65–85%) from mixed collection systems [64,65]. Contamination profiles vary substantially, with heavy metal concentrations ranging from 12 to 45 mg kg^−1^ for cadmium and 85–320 mg kg^−1^ for lead, necessitating rigorous quality control measures [51,66]. Organic fractions of municipal solid waste require sophisticated processing owing to contamination risks and variable composition. Source separation programmes significantly improve feedstock quality, with properly separated organic fractions achieving compost quality standards that meet international guidelines [67]. Processing typically involves multi-stage systems that combine mechanical separation, biological treatment, and quality control measures [68].

Advanced processing technologies encompass three primary categories of innovation in municipal solid waste treatment. First, mechanical-biological treatment (MBT) systems integrate automated sorting technologies, achieving 85–92% organic fraction recovery rates through optical recognition systems, air classification, and magnetic separation [69]. Second, negative-pressure fermentation bioreactors maintain controlled atmospheric conditions (O_2_: 12–15%, CO_2_: 8–12%) while reducing NH_3_ emissions to <1 ppm through continuous gas recirculation and biofilter treatment [70]. Third, hydrothermal carbonization processes operating at 180–250 °C and 10–40 bar pressure achieve 65–80% carbon retention while eliminating pathogens through thermal sterilisation [71].

Phosphorus pentoxide (P_2_O_5_) represents the standard reporting convention for phosphorus content in fertilisers, facilitating plant root development, ATP synthesis, and photosynthetic efficiency [72]. Potassium oxide (K_2_O) serves critical physiological functions including stomatal regulation, enzyme activation, and pathogen resistance mechanisms [73]. Primary processing challenges encompass heterogeneous composition requiring standardisation protocols, moisture-induced microbial proliferation necessitating rapid stabilisation, and heavy metal contamination demanding remediation strategies [74].

Advanced processing technologies include negative-pressure fermentation systems that reduce NH_3_ emissions to <1 ppm while maintaining optimal composting conditions [75]. These systems demonstrate 79–85% waste volume reduction, with final products achieving 1.6% nitrogen, 0.6% phosphate, and 1.4% potassium content [66]. Table 2 summarises the major characteristics of some waste feedstocks for bio-based fertiliser production.

### 2.6. Regional Variations in Feedstock Management: Nordic and Baltic Perspectives

Cold-climate regions present unique challenges and opportunities for bio-based fertiliser production. In Nordic countries and Baltic states, temperature constraints necessitate adapted processing strategies [76]. Winter temperatures below −20 °C require insulated composting systems with forced aeration to maintain thermophilic conditions, increasing operational costs by 25–35% compared to temperate regions [77].

Regional agricultural patterns generate distinctive feedstock profiles. Barley cultivation dominates in Finland (51% of cereal production), generating 1.8 million tonnes of straw annually with higher lignin content (22–26%), requiring extended processing times [78]. Forest-agricultural integration in Sweden produces mixed feedstocks combining agricultural residues with forestry wastes (bark, sawdust), achieving C:N ratio optimisation through complementary carbon and nitrogen sources [79].

Regulatory frameworks demonstrate regional specificity. The Nordic Council’s Circular Economy Programme (2021–2024) mandates 50% organic waste diversion from landfills by 2025, with specific provisions for agricultural waste valorisation [80]. Baltic states, under EU Nitrates Directive implementation, restrict winter spreading of organic fertilisers (15 October–15 April), necessitating enhanced storage capacity and stability requirements for bio-based products [81].

**Table 2:** Comprehensive characterisation of diverse agricultural and novel waste feedstocks for bio-based fertiliser production.

Feedstock Category	Specific Type	C:N Ratio	N (% Dry)	P_2_O_5_ (% Dry)	K_2_O (% Dry)	Annual Generation (Mt)	Processing Challenges	Optimal Treatment	Refs.
Cereal Residues									
Wheat straw		80–100	0.3–0.5	0.1–0.15	0.8–1.2	750	Low N content	Co-composting, pyrolysis (500–700 °C)	[13,82,83]
Rice straw		60–80	0.5–0.8	0.1–0.2	1.5–2.0	680	High lignin content	Pyrolysis, AD	[13,82,84]
Corn stover		50–60	0.6–0.9	0.2–0.3	1.2–1.5	590	Bulky material	Ensiling + AD	[85]
Barley straw		70–90	0.4–0.6	0.12–0.18	1.0–1.5	180	Variable composition	Thermophilic composting	[84]
Oilseed Residues									
Sunflower husks		45–55	1.0–1.5	0.3–0.5	2.5–3.5	8	High oil residues	Composting, Combustion	[35]
Rapeseed stalks		35–45	0.8–1.2	0.2–0.4	1.8–2.5	12	Waxy cuticle	Co-digestion with manure	[36]
Legume Biomass									
Soybean roots		20–25	2.5–3.8	0.4–0.6	1.2–1.8	45	Nodule separation	Direct incorporation	[37,86]
Pea residues		22–28	2.2–3.2	0.3–0.5	1.5–2.2	8	Rapid decomposition	Composting with bulking agent	[86]
Novel Sources									
Insect frass		8–12	2.5–4.8	1.2–2.1	1.8–3.2	0.25	Variable composition	Direct application, pelletisation	[18,34]
Buckwheat husks		50–60	0.6–0.9	0.2–0.3	0.8–1.2	1.5	High silica content	Biochar production (450–550 °C)	[38]
Food Waste									
Fruit/vegetable		15–25	2.0–3.5	0.5–0.8	2.5–3.5	510	High moisture (70–85%)	AD or composting	[87,88]
Kitchen mixed		12–20	2.5–4.0	0.8–1.2	1.5–2.5	350	Contamination risk	Source separation + AD	[74,89,90]
Animal Manure									
Cattle		15–25	1.5–2.5	0.5–1.0	1.0–2.0	1400	Pathogen presence	Thermophilic AD (55–60 °C)	[91,92]
Poultry		8–12	3.0–4.5	2.5–3.5	2.0–3.0	450	High ammonia	Composting + biochar	[56,93]
Swine		10–15	2.0–3.0	1.5–2.0	1.0–1.5	380	High water content	Solid–liquid separation	[94]
Industrial Organic									
Brewery sludge		8–12	3.5–4.5	1.5–2.0	0.3–0.5	12	Heavy metals	Co-digestion	[95,96]
Paper mill		200–400	0.2–0.4	0.1–0.2	0.1–0.2	75	Low nutrients	N supplementation	[97,98]
Sugarcane vinasse		10–15	0.3–0.5	0.1–0.2	3.5–5.0	180	High salinity	Dilution + fermentation	[22]
Municipal Organic									
Source-separated		20–30	1.5–2.5	0.5–1.0	1.0–1.5	280	Variable quality	MBT + composting	[99,100,101]
Digestate Fractions									
Liquid digestate		3–5	3.5–5.2 ^a^	0.8–1.5 ^a^	2.5–4.0 ^a^	280	High water content	Fertigation, concentration	[102,103]
Solid digestate		15–20	1.8–2.5	1.2–2.0	1.0–1.8	120	Bulky material	Soil amendment, composting	[102,103]

Note: ^a^ Concentrations in g L^−1^ for liquid fraction; Mt = Million tonnes; AD = Anaerobic Digestion; MBT = Mechanical-Biological Treatment.

### 2.7. Temporal Trends in Agricultural Waste Generation: A Decadal Assessment (2015–2025)

Statistical analysis of agricultural waste generation trajectories reveals consistent growth correlated with global agricultural intensification, population expansion, and dietary transitions toward animal-source foods. Global cereal residue production increased from approximately 2930 teragrams (Tg) in 2001 to 3900 Tg in 2020, representing a 33% cumulative increase over two decades [13]. Compound annual growth rates (CAGR) for major waste streams demonstrate differential expansion patterns: maize residues (2.0%), rice straw (1.5%), wheat straw (0.81%), and soybean residues (2.8%) [104]. Regional variations in waste generation trends reflect divergent agricultural development trajectories and policy interventions. Within the European Union, agricultural waste generation has remained relatively stable (700–720 Mt annually) over the past decade, though food waste increased to 58 million tonnes (130 kg per inhabitant) in 2023, prompting the Farm to Fork Strategy’s 50% reduction target by 2030 [105]. The EU Waste Framework Directive revisions mandate member states to report food waste data annually beginning 2020, enabling more precise temporal tracking [106].

Asian trends demonstrate contrasting patterns across major agricultural economies. China’s rice straw generation has stabilised at 270–280 Mt annually, while management practices have transformed substantially—straw return to fields increased from 46% (2009) to 52% (2019) following national burning prohibition policies [107]. India’s crop residue generation from the top 10 crops reached 686 Mt in 2018, containing 5.6 Mt of recoverable NPK nutrients, with rice-wheat systems of Punjab and Haryana generating 35 Mt of straw annually that historically underwent 80% open burning [108].

Brazilian agricultural expansion has driven dramatic increases in sugarcane-derived wastes. Production reached a record 705 Mt in 2023/24, generating 180–200 Mt of bagasse now utilised for 100% of sugar mill energy requirements, plus 360 billion litres of potassium-rich vinasse annually [22,23]. Temporal trends in agricultural waste generation over the period 2015–2024 are summarised in Table 3, which demonstrates compound annual growth rates ranging from 0.8% for rice straw to 35.2% for insect frass, reflecting divergent trajectories across feedstock categories.

## 3. Biological Treatment Technologies

### 3.1. Composting Processes and Parameters

Composting is the most widely adopted biological treatment technology, with well-established parameters for optimal performance. Aerobic composting specifications require thermophilic phases (55–65 °C) for pathogen elimination, processing durations of 60–180 days depending on feedstock, and optimal C/N ratios of 20–30:1, with final products achieving <20:1 [111].

Process optimisation strategies include aeration management through negative-pressure systems, microbial enhancement using thermophilic species (*Streptomyces thermonitrificans* and *Bacillus stearothermophilus*), and moisture control at 50–60% levels for optimal microbial activity [112]. Temperature trajectory management involves initial acidification (pH 4.3), followed by gradual increases to 7.4–8.0 in mature compost [33].

Advanced composting systems have demonstrated significant improvements in processing efficiency and product quality. Smart reactor composting systems incorporating artificial intelligence enable real-time parameter monitoring and optimisation, reducing processing times while improving the consistency of the final product [113]. These systems achieve superior pathogen elimination (>99.9%) while maintaining beneficial microbial communities [114].

### 3.2. Vermicomposting Systems and Efficiency

Vermicomposting is a specialised biological treatment that has superior product quality characteristics [115]. Processing requires 90–100 days for complete stabilisation, with careful moisture management being critical for earthworm survival [116].

Quantitative efficiency metrics demonstrate remarkable performance improvements, including 2.2–3.0-fold decreases in total organic carbon, 4.4–5.8-fold increases in total Kjeldahl nitrogen, and 79–85% waste volume reduction [117]. A meta-analysis revealed a 26% increase in commercial yield and a 78% increase in shoot biomass compared to conventional treatments [118].

Advanced vermicomposting systems include continuous flow-through reactors that enable large-scale operations with minimal labour requirements [119]. Combined treatment approaches using pre-composting followed by vermicomposting demonstrate superior stabilisation and final product quality [120]. Salt tolerance limitations require concentrations of less than 0.5% to maintain processing efficiency [121].

### 3.3. Anaerobic Digestion for Nutrient Recovery

Anaerobic digestion provides the dual benefits of energy recovery and nutrient concentration, with processing parameters optimised for different feedstock characteristics. Temperature regimes include mesophilic (35–40 °C) and thermophilic (55–60 °C) [122]. pH management maintains 6.8–7.2 for optimal methanogenesis with organic loading rates of 1–6 g VS L^−1^ day^−1^ [123].

Co-digestion optimisation using mathematical modelling determines optimal mixing ratios for food waste and livestock manure combinations, achieving biogas yields of 400–650 normal litres kg^−1^ volatile solids (VS) with methane content of 55–70% [124]. Previous studies demonstrated 250–650 L CH_4_ kg^−1^ VS processed with energy recovery of 2000 MWh year^−1^ from 6.2 million gallons of manure [125,126,127].

Advanced processing techniques include electrokinetic and ultrasonication pre-treatments, increasing ammonium-N to total N ratios in digestates, and enhancing nitrogen fertiliser replacement value by 4–14% [128]. Solid–liquid separation concentrates inorganic nitrogen in the liquid fractions (directly available) and organic nitrogen in the solid fractions (slow release) [129].

### 3.4. Fermentation Technologies and Microbial Enhancement

Fermentation technologies use specific microbial consortia for targeted waste conversion and product enhancement [130]. Fungal systems utilise *Aspergillus niger* and *Trichoderma reesei* for cellulose degradation with processing durations of 10–28 days for complete stabilisation [131].

Biofilm-based biofertilisers represent next-generation technologies containing multi-species microbial communities within protective environments to enhance competitiveness and stress tolerance [132]. These systems demonstrate superior performance under variable environmental conditions while maintaining consistent biological activity [133].

Process control innovations include real-time monitoring using IoT sensors, artificial intelligence-assisted optimisation, and automated nutrient supplementation systems [134]. These technologies enable precise control of fermentation parameters while minimising labour requirements and processing costs [135].

### 3.5. Biochar Production: Comprehensive Feedstock Utilisation

Biochar production encompasses diverse feedstock categories with distinct physicochemical outcomes. Wood industry residues constitute the largest potential feedstock, with global generation > 300 million tonnes annually [136]. Softwood bark biochar (pine, spruce) produced at 450–550 °C exhibits surface areas of 250–450 m^2^ g^−1^ with predominant microporosity (<2 nm), optimal for nutrient retention [137]. Hardwood sawdust (oak, beech) generates biochar with higher ash content (8–15%) and pH (9–11), providing enhanced liming potential [138].

Agricultural residue biochar demonstrates feedstock-specific properties. Rice husk biochar exhibits exceptional silica content (15–20%), enhancing soil water retention by 25–35% at 2% application rates [139]. Corn stover biochar, with its tubular pore structure inherited from vascular tissues, provides superior habitat for beneficial microorganisms, increasing microbial biomass by 45–65% [140]. Novel feedstocks including coffee grounds, cocoa shells, coconut husks and brewery spent grains generate biochars with elevated nitrogen content (2.5–4.5%) due to protein-rich precursors [141,142].

**Figure 1:**
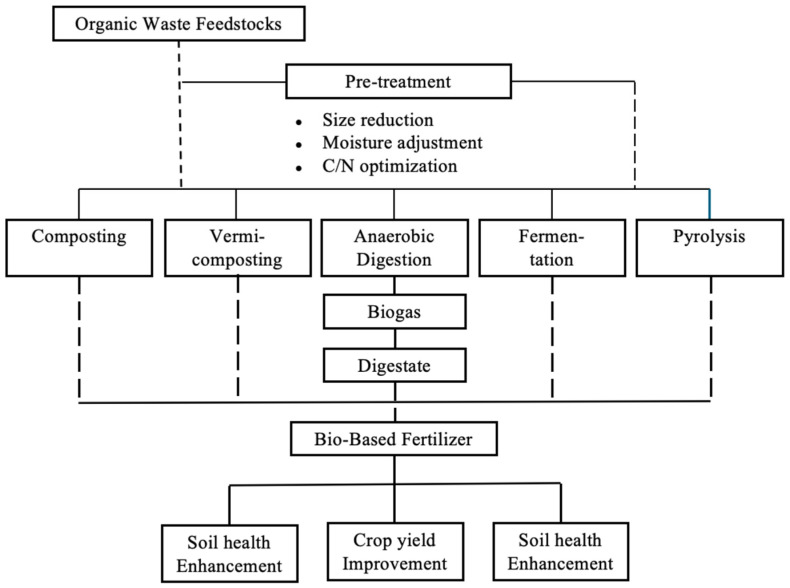
Schematic Overview of Major Biological Treatment Pathways for Organic Waste Valorization.

Pyrolysis parameters critically determine product characteristics. Slow pyrolysis (5–10 °C min^−1^) maximises char yield (25–35%), while fast pyrolysis (>100 °C min^−1^) enhances bio-oil production (50–70%) [143]. Temperature optimisation follows feedstock-specific protocols: lignocellulosic materials (500–600 °C), manures (400–450 °C), and municipal wastes (450–500 °C) [144]. Residence time variations (0.5–4 h) influence surface functionality, with extended processing enhancing aromatic carbon content and recalcitrance [145]. The alkaline pH (8–12) characteristic of biochar exhibits differential impacts on soil microbial communities. While certain alkaliphilic bacteria (*Bacillus alcalophilus*, *Alkalibacterium* spp.) demonstrate enhanced proliferation at pH 9–10, acidophilic fungi experience significant growth inhibition above pH 8 [146]. This pH-induced microbial community restructuring promotes bacterial dominance, potentially accelerating organic matter mineralization while reducing fungal-mediated carbon sequestratin [147,148]. Optimal biochar application strategies incorporate pH buffering amendments (elemental sulfur, organic acids) to maintain soil pH within the optimal range (6.5–7.5) for diverse microbial functionality [149,150]. Figure 1 shows a schematic overview of some biological treatment pathways for organic waste valorisation, and Table 4 presents a comparison of the performance of biological treatment technologies for organic waste processing.

### 3.6. Advanced Processing Technologies for Bio-Based Fertiliser Production

Beyond biological treatment, the transformation of stabilised organic materials into commercially viable fertiliser products requires sophisticated processing technologies that optimise physical properties, nutrient release kinetics, and storage stability. Two primary technological pathways dominate current research and industrial practice: granulation/pelletisation for solid bulk fertilisers and extraction/concentration for liquid fertiliser production.

#### 3.6.1. Granulation Technologies for Organic Bulk Fertilisers

Granulation converts fine organic powders, composted materials, digestates, and biochar into uniform granules (typically 2–5 mm diameter) that facilitate mechanical spreading, reduce dust generation, and enable controlled nutrient release. Four principal granulation technologies have been adapted for bio-based fertiliser production:

Drum granulation represents the predominant industrial-scale technology, employing rotating cylindrical vessels (1–3 m diameter, 4–12 m length) at rotation speeds of 9.5–17.5 r/min and inclination angles of 2–5° [155]. Granulation rates reach 70% with throughput capacities exceeding 30 tonnes per hour for large installations. A notable commercial implementation is the 30,000 tonnes/year biogas digestate granulation facility in Germany, commissioned in 2024, utilising 5 T/H drum granulator systems [156].

Disc (pan) granulation offers superior particle size uniformity through adjustable inclination (40–55°) and binder addition rates. Research by demonstrated that compressive strength varies significantly with operating parameters, with binder concentration (2–8% *w*/*w*) and pan speed (15–25 rpm) directly influencing granule integrity and average particle size distribution [157].

Extrusion granulation has emerged as an energy-efficient alternative for dry feedstocks, processing materials at 5–10% moisture without requiring post-granulation drying. Optimisation studies identified ideal parameters of 7 mm pellet diameter, 49.54 mm/min compression speed, and 7.5 MPa moulding pressure, achieving densities of 1242.49 kg/m^3^ suitable for commercial distribution [158]. Nitrogen release from extruded biocompost pellets extends to 80% over 98 days, compared to 28 days for conventional urea—demonstrating significant slow-release advantages [159].

Fluidised bed granulation enables simultaneous drying, coating, and granulation within a single unit operation, particularly advantageous for moisture-sensitive organic materials. Operating temperatures of 60–120 °C and fluidisation velocities of 1.5–3.0 m/s achieve granulation efficiencies of 65–85% with narrow particle size distributions [160]. A comparative analysis of granulation technologies for bio-based fertiliser production is presented in Table 5, encompassing operational parameters, energy requirements, capital costs, and optimal feedstock compatibility for five principal processing methodologies.

#### 3.6.2. Extraction Technologies for Liquid Fertiliser Production

Liquid bio-based fertiliser production employs extraction methodologies to solubilise and concentrate nutrients from solid organic matrices, yielding products suitable for fertigation, foliar application, and hydroponic systems. A comprehensive techno-economic assessment evaluated extraction pathways at industrial scale (300 kg/h organic waste throughput), identifying alkaline extraction as most economically viable with investment costs below €1.5 million and minimum selling prices approaching €1/L [162].

Alkaline extraction (pH 9–12, typically using NaOH or KOH) achieves nitrogen solubilisation efficiencies of 60–85% and phosphorus extraction of 40–65% from composted materials [106]. Processing parameters include solid-to-liquid ratios of 1:5 to 1:10, extraction temperatures of 40–80 °C, and residence times of 2–6 h. The resultant liquid contains humic and fulvic acids that provide plant biostimulant properties beyond macronutrient supply.

Acid extraction (pH 2–4, using H_2_SO_4_, HNO_3_, or organic acids) preferentially solubilises phosphorus from recalcitrant organic-mineral complexes. Kahiluoto et al. (2015) demonstrated that acidification increases water-extractable phosphorus in sewage sludge ashes up to 60-fold, with meat and bone meal phosphorus availability increasing from 4% to >80% when pH drops below 4 [163].

Water extraction (ambient conditions, extended contact time) produces lower-concentration but lower-cost liquid fertilisers. Vermicompost leachate (vermi-tea) production using 1:10 solid-to-water ratios and 24–48 h steeping yields products with 0.1–0.5% N, 0.05–0.2% P_2_O_5_, and 0.3–0.8% K_2_O, plus substantial microbial inoculant properties [164].

Microwave-assisted extraction represents an emerging intensification technology, reducing extraction times by 50–75% while achieving equivalent or superior nutrient recovery. Energy consumption decreases by 30–40% compared to conventional thermal extraction methods [162].

Advanced nutrient recovery technologies integrate extraction with precipitation and membrane processes to produce high-purity fertiliser products: Struvite precipitation (MgNH_4_PO_4_·6H_2_O): Achieves >95% phosphorus recovery from digestate at concentrations of 200–4000 mg P/L, with processing costs of €6.0–10.0/kg P recovered [165]; Ammonia stripping/absorption: Removes 90–97% ammonia from liquid digestate, producing ammonium sulphate solution (21% N) at costs of £4.4–4.8/kg N [166]; Membrane filtration: Reverse osmosis achieves 85–100% concentration of dissolved nutrients; nanofiltration selectively retains multivalent ions while passing monovalent species [167]; Hydrothermal carbonisation (HTC): Operating at 180–250 °C and 20 bar pressure, HTC simultaneously produces solid hydrochar (20–35% yield) and nutrient-rich process water suitable for liquid fertiliser formulation [71].

## 4. Nutrient Recovery Mechanisms and Efficiency

### 4.1. Nitrogen Recovery and Transformation

Nitrogen recovery represents the most critical aspect of BBF production because of volatilisation risks and transformation complexity. Recovery rates vary significantly by feedstock, with agricultural waste-derived BBFs achieving N content of 1.23–2.54%, with duckweed-based formulations reaching the highest concentrations [168]. Anaerobic digestion increases inorganic N content through mineralisation, with NH_4_^+^/total N ratios improving substantially during processing [102].

Pre-treatment optimisation, including electrokinetic, ultrasonication, and ensiling techniques, increases N availability by 4–14% while reducing processing times [169]. Acidification treatments prevent N losses during processing and storage by shifting the equilibrium toward non-volatile NH_4_^+^ forms [167]. Plasma treatment innovations fix reactive N from the atmosphere, creating nitrite and nitrate forms, while reducing methane and ammonia emissions during storage [170].

Advanced recovery technologies have demonstrated remarkable improvements in efficiency. Ammonia stripping and membrane separation achieve 70–95% N recovery rates in liquid digestate fractions [56]. Struvite precipitation enables simultaneous N and P recovery via crystallisation, achieving >95% nutrient extraction efficiency under optimal pH and Mg:N:P stoichiometric conditions [165].

### 4.2. Phosphorus Mobilisation and Bioavailability

Phosphorus recovery addresses critical resource scarcity and enhances agricultural sustainability. Bioavailability enhancement through acidification treatments increases water-extractable P in sewage sludge ashes up to 60-fold, with meat and bone meal P availability increasing from 4 to >80% when the pH drops below 4 [163]. BIO-peanut shells and BIO-duckweed increased soil P availability by 143.26 and 13.80%, respectively, compared to the control treatments [171].

Phosphorus placement strategies demonstrate mineral fertiliser equivalencies of 30–40% for meat/bone meal and 60–90% for untreated sewage sludge when applied using subsurface band placement (10–20 cm depth) [172]. These methods create P-rich bands that are less susceptible to surface drying, and are particularly effective in tropical soils [173].

Microbial enhancement of P solubilisation utilises specific bacterial inoculants (*Enterobacter*, *Bacillus*, and *Pseudomonas* spp.), achieving 96–99% conversion of zinc to soluble forms while enhancing overall P bioavailability [174]. These biological approaches provide sustainable alternatives to chemical acidification while maintaining long-term soil health [175].

### 4.3. Potassium Retention and Release

Potassium recovery benefits from a reduced leaching potential in BBF systems compared with synthetic alternatives. Recovery efficiency varies by processing method, with BIO-duckweed formulations containing up to 3.74% K content and achieving exchangeable K increases of 94.74% (BIO-peanut shell) and 13.08% (BIO-duckweed) over the controls [176].

Controlled-release mechanisms utilise organo-mineral combinations, where organic matrices provide physical barriers and electrostatic attraction for prolonged K retention [177]. Encapsulation technologies using chitosan-alginate matrices demonstrate sustained nutrient release, with 76.1% K initial availability, followed by gradual release patterns [178].

**Figure 2:**
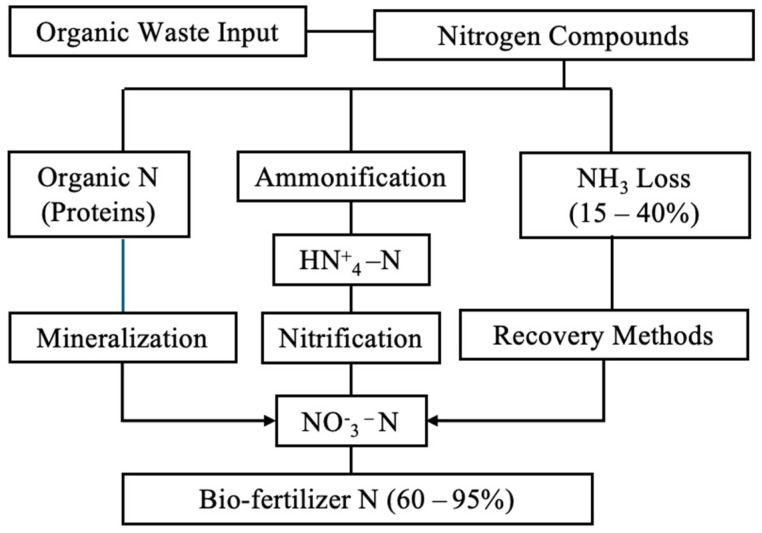
Nutrient Recovery Pathways and Transformation Mechanisms in Bio-Based Fertiliser Production.

Soil interaction dynamics showed enhanced K retention through improved cation exchange capacity in the BBF-treated soils. Organic matter additions increase CEC from typical mineral soil values of 10–100 meq/100 g to enhanced levels through organic matter contributions of 250–400 meq/100 g [179]. Table 6 shows the nutrient recovery efficiency achieved by different treatment technologies and enhancement methods, while Figure 2 shows the nutrient recovery pathways and transformation mechanisms in bio-based fertiliser production.

### 4.4. Trace Elements and Micronutrient Conservation

Trace element management requires balancing the supply of beneficial micronutrients with contamination prevention strategies. Heavy metal monitoring utilises conversion factors to predict Al, Cr, Cu, Fe, Mn, and Zn concentrations based on digestate dry matter content [180]. Quality control protocols ensure compliance with regulatory limits while maintaining the beneficial micronutrient levels [181].

Micronutrient enhancement through specific bacterial inoculants improves the bioavailability of essential elements, including Fe, Zn, Mn, Cu, B, and Mo [182]. These biological approaches provide a consistent micronutrient supply while avoiding the need for synthetic chelates [183]. Biofortification potential enables the production of specialty BBFs with enhanced micronutrient profiles for specific crop requirements [184].

### 4.5. Pathogen Reduction and Microbiological Safety

Pathogen elimination during bio-based fertiliser production requires validated treatment processes to achieve specific reduction targets [185]. Regulatory frameworks mandate the testing of indicator organisms, including *Salmonella* spp., *E. coli*, and helminth eggs, as surrogates for broader pathogenic risks [186]. Thermophilic processing, which involves achieving temperatures >55 °C for a minimum of 15 days, effectively eliminates pathogens while preserving beneficial microbial communities [187].

Molecular techniques, including qPCR and next-generation sequencing, enable comprehensive pathogen detection and microbial community characterisation [188]. Quantitative PCR (qPCR) methodologies employ sequence-specific primers targeting pathogen indicator genes, including *invA* for *Salmonella* detection (detection limit: 10^2^ CFU g^−1^), *uidA* for *E. coli* quantification (detection limit: 10^1^ CFU g^−1^), and 18S rRNA sequences for helminth egg enumeration [189]. Next-generation sequencing platforms (Illumina MiSeq, Oxford Nanopore, Oxford, UK) enable comprehensive microbial community characterisation through 16S rRNA V3-V4 region amplification (primers 341F/785R), generating >50,000 reads per sample with 97% OTU clustering for taxonomic assignment using SILVA database v138 [190]. Antibiotic resistance gene profiling utilises high-throughput qPCR arrays targeting 384 resistance genes simultaneously, quantifying absolute and relative abundances through ΔΔCt methodology normalised to 16S rRNA gene copies [191]. These advanced methods reveal antibiotic resistance gene dynamics and potential risks from emerging contaminants [192]. Quality assurance protocols incorporate regular monitoring and validation to ensure consistent pathogen reduction during production and storage [193]. While the aforementioned biological treatment technologies demonstrate substantial efficacy in organic waste valorisation, comprehensive environmental risk assessment remains paramount for ensuring product safety and regulatory compliance. Table 7 below provides an overview of the BBF environmental risk assessment of various parameters.

### 4.6. Emerging Contaminants: Microplastics and Pharmaceutical Residues

Microplastic contamination in bio-based fertilisers originates from multiple sources, with concentrations ranging from 14 to 895 particles kg^−1^ dry weight in commercial composts [194]. Primary sources include plastic mulch film fragments (polyethylene, 45–62% of total), packaging residues (polypropylene, 18–25%), and synthetic textile fibres (polyester, 12–18%) [85]. Particle size distribution analysis reveals 68% of microplastics < 1 mm, enhancing bioavailability and potential crop uptake [195].

Pharmaceutical residue persistence varies substantially across processing technologies. Tetracycline antibiotics demonstrate 35–78% reduction during thermophilic composting, with degradation rates correlating positively with temperature (r^2^ = 0.82) and inversely with initial concentration [87]. Anaerobic digestion achieves 45–92% pharmaceutical removal, with hydrophobic compounds (log Kow > 3) exhibiting higher removal efficiencies through sorption to solid matrices [88].

Biochar production at temperatures > 500 °C achieves > 99% pharmaceutical degradation through thermal decomposition, though potential formation of toxic byproducts requires assessment [89]. Risk assessment modelling indicates vegetable crops accumulate pharmaceuticals at concentrations of 0.1–15 μg kg^−1^ fresh weight when grown in amended soils, with leafy vegetables demonstrating highest uptake potential [90].

## 5. Characterisation of Bio-Based Fertilisers

### 5.1. Chemical Composition Analysis

Modern analytical approaches for BBF characterisation encompass traditional wet chemistry methods and advanced instrumental techniques. Primary nutrient analysis employs the Kjeldahl method and combustion techniques for nitrogen determination, ClO_4_-H_2_SO_4_-molybdenum-antimony colorimetric methods for phosphorus, and NH_4_OAc extraction with flame photometry, ICP-OES, or ion chromatography for potassium [197].

Advanced chemical characterisation utilises Inductively Coupled Plasma Mass Spectrometry (ICP-MS) for trace element quantification at μg kg^−1^ detection limits, X-ray Fluorescence (XRF) spectroscopy for rapid non-destructive elemental analysis, and Total Organic Carbon (TOC) analysers employing high-temperature combustion for carbon determination [198,199,200]. Standard electrode methods measure pH and electrical conductivity with 1:5 soil–water ratios, whereas gravimetric analysis at 105 °C determines moisture content [201].

Spectroscopic innovations include Fourier Transform Infrared (FT-IR) spectroscopy operating in the 4000–400 cm^−1^ range for functional group identification, with characteristic peaks at 3368 cm^−1^ (N-H, O-H stretching), 2885–2900 cm^−1^ (C-H stretching), and 1640 cm^−1^ (amide I band) [202,203]. Raman spectroscopy enables non-invasive nutrient analysis with machine learning integration for phenylpropanoid concentration analysis and real-time field capabilities [204].

### 5.2. Physical Properties Assessment

Physical property characterisation determines application compatibility and storage requirements. Bulk density and particle size measurements use standard gravimetric procedures and laser diffraction or mechanical sieving for size distribution analysis [205]. Pore size analysis provides insights into water retention characteristics, whereas infiltration rate measurements assess application efficiency [206].

Structural properties include aggregate stability measurements, porosity analysis through mercury intrusion porosimetry, and surface area determination via Brunauer–Emmett–Teller (BET) methodology [207]. These parameters directly influence the nutrient release patterns, storage stability, and application characteristics [208].

Water holding capacity evaluation uses standard laboratory methods to measure maximum water retention at field capacity, providing critical information for irrigation management and drought tolerance enhancement [209]. These measurements inform application rate recommendations and timing decisions [210].

### 5.3. Microbial Community Analysis and Functional Assessment

Microbial characterisation is a critical quality parameter that distinguishes BBFs from synthetic alternatives. 16 S ribosomal RNA (16S rRNA) gene amplicon sequencing using next-generation platforms (Illumina MiSeq, NextSeq) with primer sets F515/R806 for the V4 region enables taxonomic classification using curated databases (Greengenes, Silva, and RDP) [211].

Community structure analysis employs diversity metrics, including the Shannon-Wiener index, richness (S), and evenness (E), with beta diversity analysis revealing significant differences between treatments [212,213]. Functional analysis distinguishes total versus active communities using DNA and RNA approaches, with 25.5% bacterial and 42.3% archaeal OTUs showing significant profile differences [214].

Key microbial groups include plant growth-promoting rhizobacteria (*Enterobacter* sp., *Bacillus tequilensis*, and *Pseudomonas azotoformans*), which provide nitrogen fixation, phosphate solubilisation, and potassium mobilisation [215]. Nitrifying communities encompass ammonia-oxidising bacteria and archaea with functional redundancy, maintaining nitrogen cycling stability [216].

### 5.4. Evaluation of BBF Maturity and Stability

Stability assessment determines product readiness for agricultural applications and storage. Maturity indicators include C/N ratios < 20:1 for completed composting, respiration rates < 10 mg CO_2_-C g^−1^ organic matter day^−1^, and phytotoxicity indices > 80% for germination safety [217].

Advanced stability assessment employs differential scanning calorimetry (DSC) for thermal stability evaluation, thermogravimetric analysis (TGA) for composition determination, and pyrolysis-GC-MS for thermal decomposition product identification [218]. These techniques provide comprehensive stability profiles that are essential for product specification development [219].

Biological stability indicators include enzyme activity measurements (β-glucosidase, phosphatase, urease, and dehydrogenase), which reflect the microbial metabolic status and processing completion [220]. Pathogen monitoring ensures compliance with safety standards using quantitative PCR and cultural methods for indicator organisms [221].

### 5.5. Product Classification: Biological Versus Mineral-from-Waste Categories

Bio-based fertilisers encompass two distinct categories requiring differentiated regulatory and application frameworks. Biological products derive from microbial transformation processes, retaining organic matter matrices and active biological components. These include aerobic composts (40–60% organic matter), vermicomposts (35–50% organic matter), anaerobic digestates (solid: 60–75% organic matter; liquid: 2–5% organic matter), and fermentation products [222].

Mineral-from-waste products result from chemical precipitation, crystallisation, or extraction processes, yielding defined chemical compounds. Struvite (MgNH_4_PO_4_·6H_2_O) recovery from wastewater achieves 85–95% P recovery efficiency, producing fertiliser with 12.6% P and 5.7% N [223]. Ammonium sulfate ((NH_4_)_2_SO_4_) recovery through ammonia stripping and sulfuric acid absorption generates products with 21% N and 24% S [224]. Calcium phosphate precipitation from dairy wastewater yields products containing 18–22% P_2_O_5_ [225].

Regulatory distinctions reflect compositional differences. Biological products fall under organic fertiliser regulations (EU 2019/1009 Component Material Category 3), requiring biological stability assessment and pathogen reduction verification [226]. Mineral products classify as recovered fertilisers (CMC 12), subject to chemical purity standards and heavy metal limits [227]. Application strategies diverge accordingly: biological products optimise soil health enhancement, while mineral products target specific nutrient deficiencies [228]. Table 8 shows the analytical methods used for the bio-based fertiliser characterisation.

## 6. Effects on Soil Health

### 6.1. Physical Property Enhancement

BBFs have been shown to consistently improve soil physical properties across diverse environmental conditions. Aggregate stability increases by 35–65% following compost and digestate applications compared to unfertilised controls, with improvements occurring through increased abundance of eubacteria (+43% in compost treatments), enhanced glomalin production from arbuscular mycorrhizal fungi, and polysaccharide production by soil microorganisms [235].

Macroaggregate formation (>0.25 mm) increases with organic matter application rates, whereas microaggregates decrease, indicating improved soil structure development [236]. Water-use efficiency improvements of 54.9–176.3% in watermelon production systems demonstrate the practical benefits of enhanced soil physical properties [237].

Bulk density reduction and compaction resistance improvements result from enhanced aggregate stability and increased soil organism activity, which create a better soil structure [238]. No-till systems combined with organic amendments demonstrate superior compaction resistance, providing practical solutions for sustainable soil management [239].

### 6.2. Chemical Property Improvements

Improvements in soil chemical properties provide fundamental benefits for nutrient management and plant growth. Cation exchange capacity (CEC) increases of 15–30% following organic fertilisation result from organic matter additions with CEC values of 250–400 meq/100 g compared to 10–100 meq/100 g for mineral clays [140].

pH buffering capacity improvements enable better nutrient availability across wider pH ranges, while reducing lime requirements [240]. Enhancement in nutrient retention provides greater resistance to leaching losses, with improvements in nitrogen use efficiency of 6.9–18.5% in organic systems [241]. Phosphorus solubilisation increases through enhanced microbial activity, while sulphur mineralisation improves in organically amended soils [242].

Soil organic carbon (SOC) increases represent the most significant chemical improvement, with meta-analyses showing solid, carbon-rich bio-based fertilisers achieving 20–40% SOC increases, particularly effective in less developed soils and loamy soils in dry climates [243]. Carbon sequestration occurs through mineral-associated organic matter (MAOM) formation, increased recalcitrant organic compounds, and physical protection in soil aggregates [244].

### 6.3. Biological Property Enhancement

The biological property improvements distinguish BBFs from synthetic alternatives through enhanced soil ecosystem functionality. Microbial biomass increases of 25–65% following organic fertilisation support enhanced nutrient cycling and plant health [245]. Diversity effects from a meta-analysis of 37 studies show functional diversity 7.0% greater and bacterial/archaeal taxonomic diversity 2.9% greater in organic systems [246].

Enzyme activity enhancements include β-glucosidase (carbon cycling) increases of 45–85%, phosphatase (phosphorus cycling) increases of 30–60%, urease (nitrogen cycling) increases of 40–70%, and dehydrogenase (overall microbial activity) increases of 35–55% [247]. Increases in soil respiration of 15–40% indicate enhanced microbial activity and carbon cycling [248].

Soil fauna populations demonstrate 2–3 times higher earthworm populations with organic fertilisers, enhanced abundance and diversity of beneficial soil arthropods, and improved soil food web complexity [249]. Mycorrhizal associations show enhanced arbuscular mycorrhizal fungi colonisation, increased glomalin production, enhanced phosphorus uptake efficiency, and improved plant stress tolerance [250].

### 6.4. Long-Term Soil Health Trajectory

Long-term studies have revealed the cumulative benefits of BBF applications, which exceed the short-term nutrient supply effects. Soil quality indices consistently improve over multi-year applications, with integrated physical, chemical, and biological measurements showing 15–30% improvements compared to synthetic fertiliser systems [251].

Carbon sequestration mitigates climate change while improving soil structure and nutrient retention. Long-term carbon storage rates of 0.5–2.0 Mg C ha^−1^ year^−1^ contribute to greenhouse gas mitigation while enhancing agricultural productivity [252].

**Figure 3:**
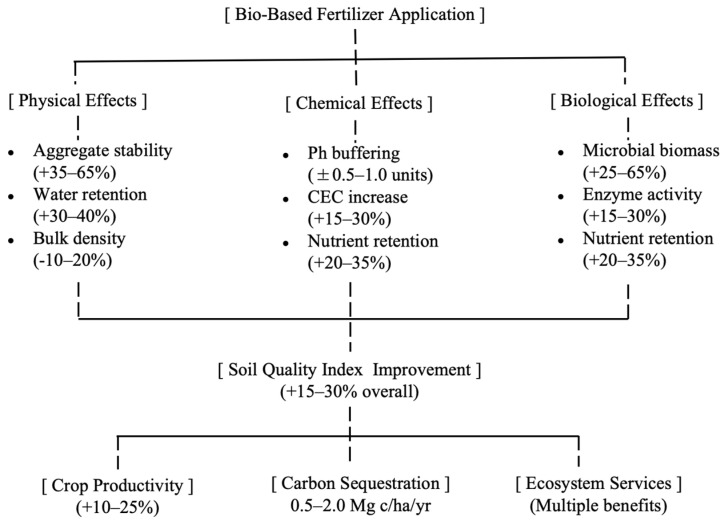
Integrated Effects of Bio-Based Fertilisers on Soil Health Parameters.

Ecosystem service provision includes enhanced water regulation, biodiversity support, and climate regulation beyond direct crop production benefits [253]. These multifunctional benefits justify premium pricing and policy support for BBF adoption [254]. Table 9 shows the meta-analysis results of the effects of bio-based fertilisers on soil properties, and Figure 3 shows the integrated effects of bio-based fertilisers.

### 6.5. Crop Yield and Quality Responses

Comprehensive yield assessments reveal that bio-based fertilisers achieve 95–112% of synthetic fertiliser yields in first-year applications, with progressive improvements over time [255]. Long-term trials spanning >5 years show yield increases of 5–56% as soil health benefits accumulate [256]. The yield response varies with crop type, soil conditions, and bio-based fertiliser quality, necessitating site-specific management strategies [257].

Crop quality parameters, including nutritional content, shelf life, and sensory attributes, often improve with bio-based fertilisation [258]. Enhanced micronutrient availability from organic sources increases crop nutritional density, while reduced chemical stress improves post-harvest quality [259]. Premium market opportunities for organically produced crops provide additional economic incentives beyond yield considerations [260].

**Table 9:** Meta-Analysis Results of Bio-Based Fertiliser Effects on Soil Properties.

Soil Property	Eff. Size (%)	Ranges	Moderating Factors	Refs.
Physical Properties				
Aggregate stability (MWD)	+42.3	35.1–49.5	Soil texture, application rate (20–40 t ha^−1^)	[236]
Water-holding capacity	+28.6	22.3–34.9	Initial SOM (<2%), climate (arid/semi-arid)	[237,238]
Bulk density	−15.2	−18.7 to −11.7	Tillage system, time (>3 years)	[236]
Infiltration rate	+35.8	28.4–43.2	Soil type (clay), management	[206]
Chemical Properties				
Soil organic carbon	+23.4	18.9–27.9	Climate (temperate), soil type (loamy)	[243,244]
Cation exchange capacity	+21.7	17.3–26.1	Clay content (<30%), OM type	[140,240]
Available N	+18.5	14.2–22.8	C:N ratio (<20:1), crop type	[241]
Available P	+31.2	25.8–36.6	pH (6.5–7.5), P-fixing capacity	[242]
pH buffering capacity	+24.3	19.8–28.8	Initial pH, lime content	[261]
Biological Properties				
Microbial biomass C	+45.3	38.7–51.9	Substrate quality, moisture	[245]
Bacterial diversity (Shannon)	+8.7	6.4–11.0	Management history, pH	[246]
Fungal–Bacterial ratio	−12.4	−15.8 to −9.0	pH increase, N availability	[262]
β-glucosidase activity	+62.4	54.2–70.6	Temperature (20–30 °C), moisture	[247]
Earthworm abundance	+156.2	128.4–184.0	Organic matter quality	[263]
Mycorrhizal colonisation	+28.9	22.5–35.3	P availability, pH	[250]

Note: MWD = Mean Weight Diameter; SOM = Soil Organic Matter.

## 7. Circular Economy Impacts and Sustainability Assessment

### 7.1. Waste Reduction and Resource Recovery Quantification

The circular economy benefits of BBFs provide quantifiable environmental and economic advantages. Global nutrient recovery potential addresses current losses of 11–14 Tg phosphorus and 120–150 Tg nitrogen annually, with recovery systems capable of reducing these losses by 60–80% through integrated processing technologies [264]. European Commission targets of 30% reduction in non-renewable fertiliser resources by 2050 demonstrate policy recognition of BBF potential [228].

**Figure 4:**
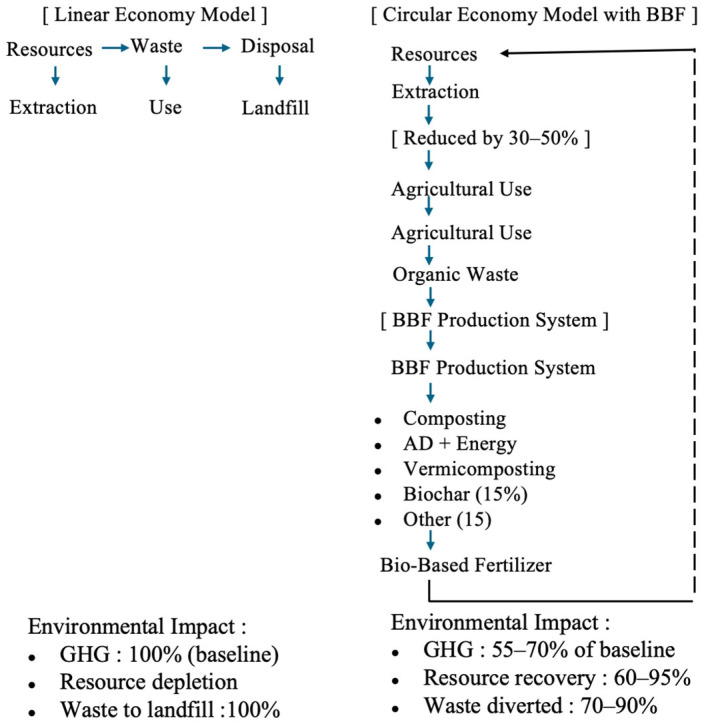
Circular Economy Framework for Bio-Based Fertiliser Systems.

Material flow analysis has revealed significant opportunities for nutrient loop closure through urban-agricultural integration. Norwegian aquaculture alone loses 66,000 tonnes of N and 14,000 tonnes of P annually to sea, equivalent to the rates of mineral fertiliser application for substantial agricultural areas [265]. Regional approaches utilising local biowastes prove more optimal than centralised facilities due to reduced transportation costs and sanitary risks [266]. Table 10 shows the life cycle assessment comparison of bio-based and synthetic fertilisers, and Figure 4 shows the circular economy framework for bio-based fertiliser systems.

### 7.2. Environmental Risk Mitigation: Nutrient Runoff and Atmospheric Emissions

Nutrient runoff from bio-based fertiliser application demonstrates substantial spatiotemporal variability. Nitrogen losses through surface runoff range from 2 to 18% of applied N, with peak losses occurring during rainfall events > 25 mm within 48 h of application [96]. Phosphorus runoff exhibits biphasic behaviour: initial soluble P release (0.5–2.5% of applied P) followed by particulate P transport (1–8% of applied P) during erosion events [97].

Mitigation strategies demonstrate variable efficacy. Incorporation of bio-based fertilisers within 24 h reduces N runoff by 45–65% and P runoff by 35–55% compared to surface application [274]. Buffer strips (5–10 m width) intercept 60–85% of nutrient runoff, with effectiveness correlating with vegetation density and hydraulic residence time [275]. Precision application technologies, utilising variable rate application based on soil nutrient mapping, reduce excess nutrient application by 20–30%, correspondingly decreasing runoff potential [276].

Gaseous emissions constitute significant environmental concerns. Ammonia (NH_3_) volatilization from bio-based fertilisers ranges from 5 to 35% of applied ammoniacal-N, with emissions influenced by pH (r^2^ = 0.76), temperature (r^2^ = 0.68), and moisture content (r^2^ = 0.52) [102]. Greenhouse gas emissions vary substantially: N_2_O emissions (0.5–3.5% of applied N), CH_4_ emissions (−2 to +15 kg ha^−1^ year^−1^), and CO_2_ emissions (500–2000 kg ha^−1^ year^−1^) [100].

Emission reduction strategies demonstrate promising results. Biochar co-application reduces N_2_O emissions by 25–45% through enhanced N immobilisation and modified microbial community composition [104]. Acidification of liquid digestates to pH 5.5–6.0 reduces NH_3_ emissions by 60–80% while maintaining nutrient availability [94]. Covered storage systems with biofilters achieve 85–95% reduction in odorous compound emissions [277].

## 8. Regulatory Frameworks and Standards

### 8.1. Global Harmonization Effects

The regulatory landscape for BBFs demonstrates increasing convergence toward harmonised standards and mutual recognition frameworks. EU Fertilising Products Regulation (FPR) 2019/1009 establishes comprehensive quality requirements, including maximum contaminant levels (cadmium < 1.5 mg/kg, lead < 120 mg/kg, mercury < 1 mg/kg dry matter), minimum nutrient specifications, and CE marking requirements for market access [226,278].

Digital innovation, through Regulation 2024/2516, introduces digital labelling options effective May 2027, modernising compliance systems and enabling real-time traceability [279]. International cooperation initiatives include G7 fertiliser supply chain stability emphasis, BRICS agricultural ministers’ discussions, and FAO Code of Conduct implementation support [280].

Quality assurance systems require ISO/IEC 17025 laboratory accreditation, with third-party certification through organisations like OMRI reviewing >10,000 products against organic standards [281]. Testing protocols encompass comprehensive chemical analysis, biological viability assessment, and environmental fate studies [282]. Table 11 presents the various international standards for bio-based fertiliser.

### 8.2. European Union Research Initiatives and Funding Programmes

The European Union has established the most comprehensive regulatory and research framework globally for bio-based fertiliser development. The Circular Bio-based Europe Joint Undertaking (CBE JU), a €2 billion public–private partnership between the European Commission and the Bio-based Industries Consortium (BIC), represents the flagship funding mechanism. Since 2014, CBE JU has invested €904 million reaching 39 countries and 1200 beneficiaries, with at least 15 projects specifically addressing bio-based fertiliser production from waste streams [286].

Key EU-funded projects include (Table 12): FERTIMANURE (Horizon 2020, €7.78 million EU grant, €8.42 million total budget, 2020–2024) deployed five on-farm pilot demonstrations across Spain, France, Germany, Belgium, and the Netherlands, producing 18 distinct bio-based fertiliser products. A critical finding: 12 of 18 products (70%) comply or can readily comply with EU Fertilising Products Regulation 2019/1009, demonstrating regulatory pathway viability [287]. SEA2LAND (€7.7 million, 2021–2024) implemented nine technologies across seven pilot sites in Baltic, Cantabric, Adriatic, North Sea, Atlantic, and Mediterranean regions, producing 15 new bio-based fertiliser products, with six exhibiting additional biostimulant properties [288]. CIRCULAR BIOCARBON (CBE JU flagship) inaugurated Europe’s first-of-its-kind municipal solid waste biorefinery in Zaragoza, Spain (October 2024), producing green graphene, bio-based fertilisers, and microalgae-derived liquid biostimulants at commercial scale [289]. LANDFEED targets production capacity of 95,000 tonnes of bio-based fertilisers annually, with projected €245.35 million sales revenue and scale-up to 580,000 tonnes within ten years of market introduction [290].

Economic analysis by Wageningen University (2025) assessed nutrient recovery potential from EU livestock manure, calculating that full implementation of struvite precipitation, ammonia stripping, and digestate processing technologies could recover 2.4 Mt nitrogen and 0.6 Mt phosphorus annually—equivalent to 35% and 45% of current EU synthetic fertiliser consumption, respectively [291].

Germany has established the most aggressive national timeline for phosphorus recovery, mandating implementation from 2029 for wastewater treatment plants serving > 100,000 population equivalents (2032 for >50,000 PE), requiring minimum 80% phosphorus recovery efficiency from sewage sludge ash or minimum 50% sewage sludge [292]. The Phosphorgewinnung Schkopau facility (Saxony-Anhalt) represents the first industrial-scale Ash2Phos plant in Germany, processing 30,000 tonnes ash annually with >90% phosphorus recovery, producing calcium phosphate fertiliser [293].

The pending RENURE criteria (REcovered Nitrogen from manURE), currently under European Nitrates Committee review, represent the next regulatory frontier. If adopted, RENURE would allow recovered nitrogen from animal manure processing to substitute for synthetic mineral fertilisers under the Nitrates Directive, with projected economic benefits including 4.8% cost reduction in livestock-intensive regions (Brittany, Lombardy, Flanders, Netherlands, Catalonia, Lower Saxony) plus 6% greenhouse gas emission reduction [294].

**Table 12:** Major International Research Programmes and Funding for Bio-Based Fertiliser Development (2020–2025).

Country/Region	Programme	Funding (Million USD/EUR)	Period	Primary Focus	Key Outputs
European Union	CBE JU (Bio-based Industries)	€904 (cumulative)	2014–present	Biorefinery integration	15+ fertiliser projects, 30+ products
EU	FERTIMANURE	€8.36	2020–2024	Manure nutrient recovery	18 BBF products, 70% regulatory compliant
EU	SEA2LAND	€9.0	2020–2024	Aquatic waste valorisation	15 BBF products, 6 biostimulants
EU	Horizon Europe calls	€150+ (fertiliser-relevant)	2021–2027	Circular nutrients	Multiple ongoing projects
United States	USDA FPEP	$900	2022–2025	Domestic production	50+ facility grants
USA	ARPA-E (various)	$50+	Ongoing	Advanced biomanufacturing	Next-gen processing
China	14th Five-Year Plan	¥5000+ (est.)	2021–2025	Green agriculture	Straw valorisation, slow-release
India	PM-PRANAM	₹25,000 crore (est.)	2023–present	Alternative fertiliser adoption	50% subsidy support
Canada	Agricultural Clean Technology	CAD $495.7	2021–2028	GHG reduction	0.8 Mt CO_2_ reduction target
Brazil	RenovaBio + state programmes	R$500+	Ongoing	Sugarcane waste integration	Vinasse-biogas-fertiliser systems

Sources: CBE JU Annual Report 2024 [289]; USDA FPEP announcements [295,296]; national agricultural ministry publications.

### 8.3. International Research Programmes and Investments

The USDA Fertilizer Production Expansion Program (FPEP) committed up to $900 million (Commodity Credit Corporation) to support domestic fertiliser production including bio-based alternatives [295]. Notable 2024 investments include $25 million to 4420 Serrano Drive LLC (California) for food waste upcycling facilities producing 11,400 tonnes of organic fertiliser annually, and $20.4 million to Myno 001 LLC (Washington) for biochar production targeting 40,000 tonnes annual output [296]. Private sector innovation has produced commercial successes, including Ostara Nutrient Recovery Technologies (Pearl^®^ Reactor System, achieving 85% P and 10–15% N recovery from wastewater), with 22+ commercial installations, including the Chicago Stickney facility—the world’s largest nutrient recovery system producing 9000 tonnes crystalline fertiliser annually [297].

Bibliometric analysis reveals China leads global sustainable fertiliser patent applications (2001–2021), with approximately 10-fold higher publication rates than other countries during 2014–2016 [298]. The 14th Five-Year National Agricultural Green Development Plan (2021) prioritises controlled-release, sustained-release, and slow-release fertiliser technologies derived from agricultural wastes. Research at Taizhou City demonstrated that integrated nutrient cycling from tangerine and water bamboo processing wastes could replace 59% nitrogen and 15% phosphorus currently supplied by synthetic fertilisers [299].

The Indian Council of Agricultural Research (ICAR) operates All India Coordinated Research Projects addressing soil nutrition and long-term fertiliser experiments, with particular focus on rice-wheat system residue management [300]. Key technologies include the Happy Seeder for rice straw incorporation (reducing particulate emissions by 78% compared to burning) and PUSA Decomposer for accelerated in-field residue breakdown. Government initiatives include PM-PRANAM (Programme for Restoration, Awareness Generation, Nourishment and Amelioration of Mother Earth), providing 50% subsidy support for bio-based fertiliser production [284].

Integration of sugarcane bagasse combustion for energy (providing 100% of sugar mill requirements) with vinasse fertigation represents the most mature commercial circular bioeconomy system globally [22,23]. Research at Federal University of São Carlos demonstrated bagasse ash sorption effectively recovers potassium and nitrogen from vinasse while neutralising pH, converting a pollution liability into a fertiliser asset [301]. São Paulo state alone possesses 66,585 MWh annual electricity generation potential from vinasse anaerobic digestion [302].

The Agricultural Clean Technology Program committed CAD $495.7 million targeting 0.8 megatonnes GHG reduction annually through clean technology adoption [303]. Specific investments include $1.69 million to Sulvaris Inc. for carbon control technology producing high-efficiency fertilisers from organic waste, and support for organic waste conversion systems serving rural communities in British Columbia [304].

## 9. Economic Viability and Market Potential

Market Growth and Competitiveness: Global BBF market analysis reveals substantial expansion from $2.53 billion (2024) to a projected $6.34 billion by 2032, representing an 8.5% CAGR across segments [305]. The regional distribution shows that North America leading with a 30–35% market share, Europe demonstrates regulatory-driven adoption, and Asia-Pacific achieves the fastest growth (12.9–13.5% CAGR), led by China and India [305].

Economic competitiveness emerges through multiple revenue streams, including tipping fees ($24–32 per tonne), energy sales from biogas production, and premium fertiliser products [306]. Cost–benefit analysis demonstrates favourable economics with <5-year payback periods for integrated systems while addressing fertilisers’ 40% share of conventional farming operating costs [307].

Investment requirements include high capital costs offset by government support mechanisms such as 50% subsidisation (India’s PM PRANAM), VAT reductions, and regulatory frameworks like EU FPR, which provide market access [284]. Business model innovation emphasises local/regional processing installations, proving more viable than centralised facilities [270]. Table 13 presents the economic analysis of BBF globally.

## 10. Challenges and Future Perspectives

### 10.1. Implementation Barriers and Solutions

Table 14 summarises the critical challenges and mitigation strategies for the implementation of bio-based fertilisers. Current barriers encompass technical challenges, including nutrient variability, processing complexity, and storage limitations, requiring advanced solutions [309]. Economic barriers involve high capital costs, market competition, and price sensitivity, which can be addressed through policy support and technology optimisation [310]. Regulatory complexity, including approval processes, standard inconsistency, and quality requirements, necessitates harmonisation efforts and streamlined pathways [278]. Market acceptance challenges regarding farmer preferences and consumer concerns require education, demonstration programmes, and quality assurance [311].

### 10.2. Emerging Technologies and Innovations

Next-generation technologies include smart controlled-release systems using biosensors that respond to plant signalling molecules, biofilm-based biofertilizers with multi-species communities, and nano-bioconjugates that combine nanotechnology with biological systems [312]. Manufacturing innovations encompass 3D printing for customised pellets, green chemistry biodegradable coatings, and AI-driven formulation optimisation [313]. Precision agriculture integration utilises IoT sensor networks for real-time monitoring, variable rate application systems, satellite-based optimisation, and digital twin technologies for predictive management [314]. Climate adaptation incorporates temperature-responsive release, pH buffering enhancement, and multi-stressor protection capabilities [315].

## 11. Conclusions

This comprehensive review synthesises current understanding of bio-based fertiliser production from organic waste streams within circular economy frameworks. The analysis demonstrates that biological treatment technologies—encompassing composting, vermicomposting, anaerobic digestion, and thermochemical conversion—constitute viable pathways for transforming organic residues into valuable agricultural inputs while simultaneously addressing waste management imperatives.

Meta-analytical evidence confirms significant enhancement of soil physical, chemical, and biological properties following bio-based fertiliser application, with documented improvements in aggregate stability, nutrient retention, and microbial diversity substantiating multifunctional benefits beyond nutrient provision. Life cycle assessments consistently demonstrate reduced greenhouse gas emissions relative to synthetic fertiliser production, supporting climate change mitigation objectives.

Critical challenges persist, including regulatory heterogeneity across jurisdictions, emerging contaminant management, and economic optimisation requirements. The substantial variance in international standards, particularly regarding heavy metals and microplastic contamination, necessitates harmonised assessment protocols. Future research priorities should emphasise emerging contaminant quantification, predictive modelling of treatment outcomes, and longitudinal assessment of cumulative soil health impacts.

The transition toward sustainable agricultural intensification requires fundamental reconceptualization of organic waste as a renewable resource. Bio-based fertilisers represent an essential nexus linking waste valorisation, agricultural productivity, and environmental protection. Successful implementation demands coordinated stakeholder engagement, continued technological innovation, and evidence-based policy formulation to realise the transformative potential of circular bioeconomy paradigms in agricultural systems.

## Figures and Tables

**Table 1:** Global Agricultural Waste Generation by Category, Top Producing Countries, and Annual Quantities (2020–2024).

Waste Category	Global Annual Production (Mt)	Top Producer (Mt)	Second Producer (Mt)	Third Producer (Mt)	Utilisation Rate (%)
Cereal Residues					
Corn stover	1100–1200	USA (250–364)	China (216–220)	Brazil (85–95)	35–40
Wheat straw	750–850	China (140–150)	India (100–110)	Russia (55–60)	45–55
Rice straw	731	China (270+)	India (130–140)	Indonesia (65–70)	25–35
Barley straw	180–200	Russia (35–40)	Canada (25–30)	Germany (18–22)	50–60
Oilseed Residues					
Sunflower residues	60–70	Russia (17–20)	Ukraine (14–18)	Argentina (8–10)	15–25
Rapeseed stalks	45–55	China (12–15)	Canada (10–12)	EU-27 (8–10)	30–40
Soybean residues	350–400	USA (95–110)	Brazil (85–95)	Argentina (45–55)	40–50
Leguminous Residues					
Lupine residues	3–5	Australia (1.8–2.2)	Poland (0.4–0.5)	Germany (0.15–0.2)	20–30
Pea/bean residues	25–35	Canada (5–7)	Russia (4–5)	France (3–4)	35–45
Specialty Crops					
Buckwheat residues	2–3	Russia (1.15–1.22)	China (0.5–0.6)	Ukraine (0.1–0.15)	10–20
Rice husks	74	China (22–25)	India (18–20)	Indonesia (8–10)	<20
Sugarcane bagasse	180–200	Brazil (95–105)	India (35–40)	China (18–22)	85–95
Animal Manure					
Cattle manure	4550	India (850–900)	Brazil (650–700)	China (550–600)	60–70
Poultry manure	770	China (180–200)	USA (150–170)	Brazil (80–90)	70–80
Swine manure	520	China (280–300)	EU-27 (85–95)	USA (65–75)	65–75
Novel Waste Streams					
Insect frass (BSF)	0.25–0.35 ^a^	EU-27 (0.08–0.1)	USA (0.05–0.07)	Asia (0.04–0.06)	>90

Note: ^a^ Projected to reach 1.2 million tonnes by 2030; Mt = Million tonnes; BSF = Black Soldier Fly; Utilisation rate refers to proportion directed toward agricultural or energetic valorisation rather than open burning or landfilling. Compiled from FAO Statistical Yearbook 2024 [16]; Shah et al. [17]; Lal, 2005 [13]; Beesigamukama et al., 2020 [18].

**Table 3:** Temporal Trends in Agricultural Waste Generation: Five-Year Comparative Analysis (2015–2024).

Feedstock Category	2015 Estimate (Mt)	2020 Estimate (Mt)	2024 Estimate (Mt)	CAGR 2015–2024 (%)	Primary Drivers
Cereal Residues					
Total cereal residues (Global)	3500	3900	4100	1.8	Yield intensification, area expansion
Rice straw	680	710	731	0.8	Stable area, yield gains
Wheat straw	720	750	780	0.9	Production expansion in Russia, Ukraine
Corn stover	1450	1580	1661	1.5	US/Brazil/China expansion
Oilseed Residues					
Sunflower residues	48	58	65	3.4	Black Sea region expansion
Soybean residues	280	340	385	3.6	Brazil expansion, China demand
Specialty Crops					
Buckwheat residues	1.8	2.2	2.5	3.7	Health food market growth
Sugarcane bagasse	155	175	195	2.6	Ethanol expansion (Brazil)
Animal Manure					
Total livestock manure	8200	8800	9260	1.4	Global livestock population growth
Poultry manure	650	720	770	1.9	Intensive poultry production
Novel Streams					
Insect frass	0.02	0.08	0.30	15–28	EU novel food approvals, investment
Food Waste					
Global food waste	930	1050	1300	3.8	Urbanisation, supply chain losses

Note: CAGR = Compound Annual Growth Rate. Sources: FAO Statistical Yearbook 2024 [16]; FAOSTAT crop production databases [109]; Kaza et al., 2018 [64]; UNEP Food Waste Index 2024 [110].

**Table 4:** Comparative Performance Metrics of Biological Treatment Technologies for Organic Waste Processing.

Technology	Duration	Tem.	C/N Final	Nutrient Recovery (%)	Vol. Reduction (%)	Energy Balance	Product Quality Assessment	Refs.
Aerobic Composting	35–120 days	55–65 °C	<20:1	N: 40–60, P: 80–90, K: 85–95	50–65	Negative (aeration required)	Maturity: C:N < 20:1 GI > 80% Stable, pathogen-free	[33,111,112,113,114]
Vermicomposting	90–100 days	20–30 °C	12–15:1	N: 70–85, P: 85–95, K: 90–98	79–85	Neutral	Maturity: OM > 40% pH 6.5–8.0 High biological activity	[115,116,118,120]
Mesophilic AD	20–40 days	35–40 °C	Variable	N: 60–80, P: 90–95, K: 95–98	40–50	Positive (biogas: 400–550 NL kg^−1^ VS)	CH_4_: 55–65% VS reduction > 50% Liquid and solid fractions	[122,123,124,125]
Thermophilic AD	15–25 days	55–60 °C	Variable	N: 65–85, P: 90–95, K: 95–98	45–55	Positive (biogas: 450–650 NL kg^−1^ VS)	CH_4_: 60–70% Pathogen-free Higher NH_4_^+^/TN ratio	[122,123,124,125,128,129]
Fermentation	10–28 days	25–45 °C	15–25:1	N: 50–70, P: 75–85, K: 80–90	30–45	Negative	Enhanced microbial content Biofilm formation Enzyme activity	[130,133,134,135]
Pyrolysis	0.5–2 h	300–1000 °C	High C	N: 20–40, P: 70–90, K: 80–95	70–85	Variable (depends on energy recovery)	Stable carbon (40–90%) Alkaline pH (8–12) Surface area: 100–800 m^2^ g^−1^	[144,151,152,153,154]

Note: GI = Germination Index; OM = Organic Matter; VS = Volatile Solids; NL = Normal Litres; TN = Total Nitrogen.

**Table 5:** Comparative Performance of Granulation Technologies for Bio-Based Fertiliser Production.

Technology	Capacity (t/h)	Granule Size (mm)	Moisture Req. (%)	Energy (kWh/t)	Capital Cost (€/t capacity)	Optimal Feedstock	Refs.
Drum granulation	5–30	2–8	25–45	15–25	150–250	Digestate, compost	[155,156]
Disc (pan) granulation	1–15	1–5	20–35	20–35	200–350	Fine powders, biochar blends	[157]
Extrusion (screw press)	0.5–5	4–10	5–15	25–40	250–400	Dry compost, manure	[158,159]
Fluidised bed	1–10	0.5–3	Variable	40–60	400–600	Coating applications	[160]
Roll compaction	2–20	2–6	<10	30–45	300–450	Ash, mineral blends	[161]

Note: t/h = tonnes per hour; kWh/t = kilowatt-h per tonne.

**Table 6:** Nutrient Recovery Efficiency by Treatment Technology and Enhancement Methods.

Technology	Base Rec. (%)	Enhancement Method	Tem. (°C)	Time	Substrate	Enhanced Rec. (%)	Key Mechanisms	Refs.
Nitrogen Recovery								
Composting	40–60	Bulking agents (woodchips, biochar)	55–65	35–45 days	Mixed organic waste	55–70	Reduced NH_3_ volatilization through adsorption	[102,168]
AD-Mesophilic	60–75	Acidification to pH 5.5–6.0	35–40	20–30 days	Food waste + manure (1:1)	75–85	pH control optimises methanogenesis	[169]
AD-Thermophilic	65–80	Plasma treatment	55–60	15–25 days	Agricultural residues	80–95	Atmospheric N fixation, reactive N species	[170]
Stripping + Absorption	85–90	Membrane separation (hollow fibre)	60–80	2–4 h	Liquid digestate	90–95	Selective NH_3_ permeation	[56,165]
Phosphorus Recovery								
Direct application	30–40	Acidification (H_2_SO_4_, pH < 4)	Ambient	24 h	Sewage sludge ash	60–80	P solubilization from Ca-P complexes	[163,171]
Struvite precipitation	80–90	Mg supplementation (MgCl_2_, MgO)	20–25	30 min	Wastewater	90–95	Crystal formation at pH 8.5–9.0	[172,173]
Microbial solubilization	50–60	Specific inoculants (*Bacillus*, *Pseudomonas*)	28–32	7–14 days	Rock phosphate	70–85	Organic acid production (citric, oxalic)	[174,175]
Potassium Recovery								
Composting	85–90	Moisture control (50–60%)	55–65	35–45 days	Agricultural waste	90–95	Leaching prevention through water management	[176]
Anaerobic digestion	90–95	Solid retention (screw press)	35–55	20–40 days	Mixed feedstock	95–98	Ion exchange with organic matrix	[177,178]
Biochar production	80–90	Lower temperature (400–450 °C)	400–450	1–2 h	Manure-based	85–95	Reduced volatilization, ash retention	[179]

**Table 7:** Environmental Risk Assessment Parameters for Bio-Based Fertilisers.

Contaminant Category	EU Limit Values	USEPA St.	Risk Assessment Methods	Mitigation Strategies	Monitoring Frequency	Refs.
Heavy Metals (mg kg^−1^ DM)						
Cadmium (Cd)	1.5	39	ICP-MS, XRF, AAS	Source control, pH adjustment (>6.5)	Quarterly	[74,91]
Lead (Pb)	120	300	ICP-OES, AAS	Chelation, phytoremediation	Quarterly	[91,92]
Mercury (Hg)	1.0	17	CV-AAS, ICP-MS	Thermal treatment (>350 °C)	Bi-annual	[92]
Chromium (Cr)	100	1200	ICP-MS, XRF	Reduction (Cr^6+^ to Cr^3+^)	Quarterly	[74]
Arsenic (As)	40	41	HG-AAS, ICP-MS	Iron co-precipitation	Quarterly	[74]
Organic Pollutants						
PAHs (Σ16)	6 mg kg^−1^	3 mg kg^−1^	GC-MS, HPLC-FLD	Extended composting (>120 days)	Annual	[93]
PCBs (Σ7)	0.8 mg kg^−1^	1.0 mg kg^−1^	GC-ECD, GC-MS	Biodegradation, thermal treatment	Annual	[56]
Dioxins/Furans	30 ng TEQ kg^−1^	50 ng TEQ kg^−1^	HRGC-HRMS	Source exclusion	Bi-annual	[56]
Emerging Contaminants						
Microplastics	No standard	No standard	FTIR, Raman, py-GC-MS	Source separation, screening	Research phase	[85,194,195]
Pharmaceuticals	Under development	Variable	LC-MS/MS, UPLC-QToF	Advanced oxidation, biochar	Research phase	[87,88,89]
Antibiotics	<1 mg kg^−1 a^	Variable	LC-MS/MS	Thermophilic treatment	Quarterly	[87]
Biological Hazards						
*Salmonella* spp.	Absent/25 g	<3 MPN/4 g	Culture, qPCR, LAMP	Time-temperature (55 °C, 3 days)	Each batch	[95,196]
*E. coli*	<1000 CFU g^−1^	<1000 MPN g^−1^	Selective media, qPCR	PFRP compliance	Each batch	[95]
Helminth eggs	<1 viable egg/4 g	<1 viable egg/4 g	Microscopy, flotation	Alkaline treatment (pH > 12)	Monthly	[196]
Nutrient Runoff Risk						
Total N	Application limits	State-specific	Soil testing, modelling	Split application, inhibitors	Pre-application	[96,97]
Total P	P-index based	State P-index	Soil P saturation	Buffer strips, incorporation	Pre-application	[97,98]
Gaseous Emissions						
NH_3_	<20% of TAN	Variable	Acid traps, sensors	Acidification, incorporation	During application	[94,99]
N_2_O	<1% of applied N	No standard	Chamber methods, GC	Biochar, inhibitors	Seasonal	[100,101]
CH_4_	<10 kg ha^−1^ yr^−1^	No standard	Chamber methods, GC	Aerobic conditions	Seasonal	[100]

Note: ^a^ Proposed limit; DM = Dry Matter; TEQ = Toxic Equivalents; TAN = Total Ammoniacal Nitrogen; PFRP = Process to Further Reduce Pathogens; LAMP = Loop-mediated Isothermal Amplification.

**Table 8:** Analytical Methods for Bio-Based Fertiliser Characterisation.

Parameter Category	Analytical Method	Detection Range	Standards	Advantages	Limitations	Refs.
Chemical Analysis						
Total N (%)	Kjeldahl/Combustion	0.1–10	ISO 11261:1995	Accurate, established method	Time-consuming (4–6 h)	[82,84]
Available P (mg kg^−1^)	Olsen/Bray methods	1–500	ISO 11263:1994	Crop-relevant assessment	pH-dependent extractionb	[83]
Exchangeable K (mg kg^−1^)	NH_4_OAc extraction	10–5000	ISO 11260:2018	Standard method, reproducible	Matrix effects possible	[197]
Organic matter (%)	Loss on ignition	1–100	ASTM D2974	Simple, rapid (2–4 h)	Carbonates interference	[229]
Heavy metals (mg kg^−1^)	ICP-MS/ICP-OES	0.01–1000	EPA 3051A	Multi-element analysis	Matrix interference	[74]
Physical Properties						
Particle size (μm)	Laser diffraction	0.01–3000	ISO 13320:2020	Rapid, automated	Sample preparation critical	[230]
Bulk density (g cm^−3^)	Core method	0.1–1.8	ISO 11272:2017	Direct measurement	Disturbed samples	[230]
Water holding (%)	Pressure plate	0–100	ISO 11274:2019	Multiple pressure points	Time-intensive (24–48 h)	[205]
Biological Assessment						
Microbial biomass (mg C kg^−1^)	Fumigation-extraction	50–5000	ISO 14240-2:1997	Sensitive to change	Extraction efficiency variable	[85,194]
Enzyme activities (μg g^−1^ h^−1^)	Substrate assays	Variable	ISO 20130:2018	Functional indicator	Temperature sensitive	[231]
16S rRNA sequencing	NGS platforms (Illumina)	>10^4^	QIIME2 protocols	Comprehensive profiling	Bioinformatics required	[190]
Pathogen detection (CFU g^−1^)	qPCR/Culture	10–10^6^	ISO 19250:2010	Specific, sensitive	Viable cells only	[95,196]
Spectroscopic Methods						
FT-IR	Fourier Transform IR	4000–400 cm^−1^	ASTM E1252	Non-destructive, functional groups	Qualitative/semi-quantitative	[202,232]
XRD	X-ray Diffraction	2θ: 5–90°	ISO 13925-3:2015	Crystal structure identification	Amorphous materials limited	[233]
NMR	Nuclear Magnetic Resonance	^13^C, ^31^P, ^15^N	Literature methods	Detailed molecular structure	Expensive, complex	[234]

**Table 10:** Life Cycle Assessment Comparison of Bio-Based vs. Synthetic Fertilisers.

Impact Category	Unit	Synthetic NPK	Composting	Anaerobic Digestion	Biochar		Impact Reduction (%)	Refs.	
Environmental Impacts									
Global warming potential	kg CO_2_-eq kg^−1^ N	8.2–10.5	2.8–4.2	1.5–3.2	2.1–3.8		45–80	[267,268,269]	
Acidification potential	kg SO_2_-eq kg^−1^ N	0.042	0.018	0.015	0.012		57–71	[264]	
Eutrophication potential	kg PO_4_-eq kg^−1^ N	0.018	0.008	0.006	0.005		56–72	[270]	
Energy consumption			MJ kg^−1^ N	45–60	8–15	−5 to 10 ^a^	10–20	67–108	[265]
Water consumption			L kg^−1^ product	85–120	20–40	15–30	10–25	67–88	[266]
Resource Recovery									
N recovery from waste	%	0	40–60	60–85	20–40		+40–85	[56,102]	
P recovery from waste	%	0	80–90	90–95	70–90		+70–95	[163,172,173,174,175]	
K recovery from waste	%	0	85–95	95–98	80–95		+80–98	[176,177,178,179]	
Organic matter recycled			kg ton^−1^ waste	0	150–250	100–200	200–300	+100–300	[243,244]
Carbon sequestration			kg C ton^−1^ product	−50 to −80 ^b^	50–100	30–60	200–400	+250–480	[271,272,273]

Note: ^a^ Negative values indicate net energy production through biogas; ^b^ Negative values indicate carbon emissions.

**Table 11:** International Regulatory Standards for Bio-Based Fertilisers.

Region/ Country	Regulation	Key Requirements	Heavy Metal Limits (mg kg^−1^ dry matter)	Pathogen Standards	Implementation	Refs.
European Union	Regulation 2019/1009	CE marking, PFC categories	Cd: <1.5–3, Pb: <120, Hg: <1, Cr(VI): <2	Salmonella: absent/25 g E. coli: <1000 CFU g^−1^	July 2022	[278]
United States	EPA 503, State regulations	Class A/B biosolids	Cd: <39, Pb: <300, Hg: <17, As: <41	Faecal coliform: <1000 MPN g^−1^	Varies by state	[283]
Canada	CFIA T-4-93 to T-4-130	Registration required	Cd: <20, Pb: <500, Hg: <5, As: <75	Product-specific	Ongoing	[280]
China	GB/T 23349-2020	Organic matter > 45%	Cd: <3, Pb: <50, Hg: <2, As: <15, Cr: <150	Faecal coliform: <100 MPN g^−1^	2020	[282]
India	FCO 1985 (amended)	Minimum nutrients specified	Cd: <5, Pb: <100, Hg: <0.15, As: <10	Total coliforms: <1000 MPN/g	Ongoing updates	[284]
Australia	AS 4454-2012	Composting standards	Cd: <1–20 *, Pb: <150–420 *, Hg: <1–4 *	Varies by grade	2012 revised	[285]
ISO Standards	ISO 17025:2017	Laboratory accreditation	Method-specific	Method validation	Global adoption	[282]

* Varies by contamination grade classification (A, B, C grades).

**Table 13:** Economic Analysis of Bio-Based Fertiliser Production Systems.

Production System	Capital Cost ($ ton^−1^ Capacity)	Operating Cost ($ ton^−1^)	Revenue Streams	Payback Period (Years)	IRR (%)	NPV (20 Year, Million $)	Refs.
Small-Scale Systems (<10,000 tons year^−1^)							
Windrow composting	50–150	25–40	Product sales	4–7	12–18	0.5–2.0	[306,307]
Vermicomposting	100–250	35–55	Product + worms	3–5	15–25	1.0–3.0	[270]
Farm-scale AD	300–500	20–35	Energy + fertiliser	5–8	10–15	2.0–5.0	[125]
Medium-Scale Systems (10,000–50,000 tons year^−1^)							
In-vessel composting	200–400	30–45	Product + tipping fees	3–5	18–25	5.0–15.0	[306]
Centralised AD	400–700	25–40	Energy + digestate + RECs	4–6	15–22	10.0–25.0	[122]
Biochar production	250–450	40–60	Biochar + carbon credits	5–7	12–20	3.0–8.0	[151]
Large-Scale Systems (>50,000 tons year^−1^)							
Industrial composting	150–300	20–35	Multiple products	3–4	20–30	20.0–50.0	[270]
Municipal AD + upgrading	500–900	30–50	Biomethane + fertiliser	5–7	18–28	30.0–80.0	[266]
Integrated biorefinery	800–1200	35–55	Multiple value streams	6–8	15–25	50.0–150.0	[308]

Note: IRR = Internal Rate of Return (percentage return on investment); NPV = Net Present Value (discounted cash flow over project lifetime); RECs = Renewable Energy Certificates; AD = Anaerobic Digestion.

**Table 14:** Critical Challenges and Mitigation Strategies for Bio-Based Fertiliser Implementation.

Challenge Category	Specific Issues	Impact Level	Current Solutions	Emerging Technologies	Research Priorities	Refs.
Technical Challenges						
Nutrient variability	Batch inconsistency (CV: 15–40%)	High	Standardisation, blending	NIR spectroscopy, AI optimisation	Real-time monitoring	[309]
Processing complexity	Multiple unit operations	Medium	Integrated systems	Automated control systems	Process intensification	[310]
Storage stability	Degradation, moisture uptake	Medium	Controlled environment	Smart packaging, sensors	Stabilisation additives	[278]
Pathogen control	Survival in products	High	Thermal treatment (>55 °C, 15 days)	Plasma treatment, UV-C	Rapid detection methods	[221]
Economic Barriers						
Capital intensity	$200–900 ton^−1^ capacity	High	Government subsidies (20–50%)	Modular systems	Cost reduction pathways	[306]
Market competition	Price vs. synthetic (1.2–2.5×)	High	Premium markets	Value-added products	Differentiation strategies	[307]
Scale economies	Small producer disadvantage	Medium	Cooperatives	Distributed processing	Business model innovation	[270]
Regulatory Issues						
Standards inconsistency	Regional variations (26× for Cd)	High	Harmonisation efforts (EU, ISO)	Digital compliance	Global frameworks	[278,281,282]
Approval timelines	2–5 years for new products	Medium	Fast-track pathways	Risk-based assessment	Streamlined procedures	[278]
Quality assurance	Testing requirements ($500–5000/batch)	Medium	Accredited labs	Rapid test methods	Standardised protocols	[282]
Market Acceptance						
Farmer scepticism	Performance concerns	High	Demonstration trials (>100 sites)	Precision agriculture	Extension programmes	[311]
Consumer perception	Safety concerns	Medium	Certification schemes (OMRI, EU)	Transparent labelling	Education campaigns	[311]
Supply chain integration	Logistics challenges	Medium	Hub-and-spoke models	Digital platforms	Infrastructure development	[266]

Note: CV = Coefficient of Variation; OMRI = Organic Materials Review Institute.

## Data Availability

No new data were created for this review.
